# Epidemiology of Neuro-Behçet’s Disease in Northern Spain 1999–2019: A Population-Based Study

**DOI:** 10.3390/jcm13175270

**Published:** 2024-09-05

**Authors:** Alba Herrero-Morant, José Luis Martín-Varillas, Carmen Álvarez-Reguera, Lara Sánchez-Bilbao, David Martínez-López, Guillermo Suárez-Amorín, Raúl Fernández-Ramón, Iván Ferraz-Amaro, Santos Castañeda, José L. Hernández, Ricardo Blanco

**Affiliations:** 1 Rheumatology Division, Hospital Universitario Marqués de Valdecilla, Immunopathology Group-IDIVAL, Avda. Valdecilla s/n., 39008 Santander, Cantabria, Spain; 2 Rheumatology, Hospital de Laredo, 39770 Laredo, Cantabria, Spain; 3 Ophthalmology, Hospital Universitario Marqués de Valdecilla, Immunopathology Group-IDIVAL, Avda. Valdecilla s/n., 39008 Santander, Cantabria, Spain; 4 Rheumatology, Hospital Universitario de Canarias, 38320 La Laguna, Tenerife, Spain; 5 Rheumatology, Hospital Universitario La Princesa, IIS-Princesa, 28006 Madrid, Spain; 6 Internal Medicine, Hospital Universitario Marqués de Valdecilla, Immunopathology Group-IDIVAL, Avda. Valdecilla s/n., 39008 Santander, Cantabria, Spain

**Keywords:** epidemiology, Behçet’s disease, neurologic manifestations

## Abstract

**Background/Objectives**: Neuro-Behçet’s disease (NBD) is one of the most severe complications of Behçet’s disease (BD). The incidence of NBD varies widely worldwide. This study aimed to estimate its current incidence in Northern Spain. **Methods**: This was a retrospective population-based cohort study of 120 patients in Northern Spain diagnosed with BD according to the 2013 International Criteria for BD (ICBD) between 1 January 1999 and 31 December 2019. NBD diagnoses were made according to International Consensus Recommendation (ICR) criteria. Overall, 96 patients were included, and their demographic and clinical data were collected. The incidence of NBD was estimated by age, gender, and year of diagnosis between 1999–2019. **Results**: NBD was diagnosed in 23 of 96 (24%) patients (15 women/8 men) (mean age: 44 ± 13.9 years). HLA-B51 was positive in 5 of 13 (38.4%) cases tested. A total of 10 (43.5%) patients had parenchymatous NBD, 10 (43.5%) had non-parenchymatous NBD, and 3 (13%) had mixed NBD. Incidence during the study period was 0.13 (95% CI, 0.11–0.26) per 100,000 people-years. There were no significant differences in gender in the incidence rate stratified by age (*p* > 0.05). Furthermore, there was a linear relationship with a mild decrease in age at diagnosis over time. **Conclusions**: Epidemiological characteristics of NBD in Northern Spain are similar to those of neighboring countries, except female gender predominance.

## 1. Introduction

Behçet’s disease (BD) is a systemic, chronic relapsing inflammatory disease, typically characterized by recurrent oro-genital ulcers, ocular inflammation, and skin, joint, vascular, gastrointestinal, and neurological involvement [1,2,3]. Neuro-BD (NBD) is a complication of BD, including neurological manifestations of the disease, and encompasses some of the most severe presentation forms [3,4].

The epidemiology of BD presents geographical differences, with a higher prevalence in ancient Silk Road countries [5]. Regional variability is a well-known epidemiological feature of BD [6]. A complex genetic and environmental background may contribute to the disease’s development, incidence, and individual symptoms [1,6].

The incidence of neurological involvement in BD varies between studies and is associated with high morbimortality [7]. Nevertheless, epidemiological studies mainly focus on BD rather than NBD [5,8]. Data gathered for BD seem to be consistent with NBD etiopathogenesis [5]. 

Taking all these considerations into account, we present, to the best of our knowledge, the most extensive Spanish series of patients with NBD. This study aimed to characterize NBD epidemiology, focusing on clinical characteristics and annual incidence, in a well-defined cohort of BD patients diagnosed between 1999 and 2019 in Cantabria, a region located in Northern Spain (Figure S1).

## 2. Materials and Methods

### 2.1. Design and Enrollment Criteria

We conducted a retrospective cohort study of 120 patients diagnosed with BD according to expert opinion criteria between 1 January 1999 and 31 December 2019. Patients were subclassified according to the 2013 International Criteria for Behçet’s Disease (ICBD) [9]. This study was carried out at the three main hospitals in the Cantabria Region in the north of Spain: Hospital Universitario Marqués de Valdecilla, Hospital de Sierrallana, and Hospital de Laredo. Patients were identified through the intranet resources of the Cantabrian Health Service: the hospitals’ diagnostic coding system, the International Classification of Primary Care (CIAP) used in the health centers, and data provided by different departments (Pneumology, Ophthalmology, Dermatology, Neurology, Internal Medicine, Nuclear Medicine, Pathological Anatomy, Radiology, and Rheumatology). Ninety-six BD patients living in Cantabria were finally included in this study. This study was approved by the Clinical Research Ethics Committee (ethical approval code: 2020.083).

Diagnoses of NBD were based on the International Consensus Recommendation (ICR) criteria for NBD diagnosis [4]. A diagnosis of “definite NBD” was established if the criteria for BD were fulfilled and the patient had a neurological syndrome (with objective neurological signs) based on relevant and characteristic abnormalities of NBD observed in either neuroimaging or cerebrospinal fluid, or both [9]. There was no other explanation for neurological findings [9]. Patients with “definite NBD” were classified according to the consensus classification of NBD in the central nervous system (CNS) and peripheral nervous system involvement [4]. In addition, CNS involvement was subclassified as parenchymal, non-parenchymal, or mixed disease according to clinical, laboratory, neuro-radiological, pathological, and prognostic characteristics [4].

### 2.2. Outcome Variables

The following variables were retrospectively obtained from medical histories: demo-graphic data (gender and age at diagnosis), NBD phenotype (parenchymal, non-parenchymal, or mixed involvement), HLA-B51 presence, organ involvement distributed according to the EULAR recommendations [10], and treatment. 

### 2.3. Data Collection and Statistical Analysis

Outcome variables were documented at each center following a pre-established clinical protocol. Data were entered into a computerized database, and all information was thoroughly verified to reduce the risk of input errors.

Population data were sourced from the annual reports of the Cantabria Health Service (https://www.scsalud.es/memorias (accessed on 15 December 2020) and the National Statistics Institute (https://www.ine.es/ (accessed on 15 December 2020) to estimate incidence rates. The annual incidence rate was determined by calculating the number of new NBD cases as a proportion of the at-risk population for each corresponding year. Additionally, analyses of NBD’s average annual incidence rate and main general features were conducted at 5-year intervals from 1999 to 2019.

Results were presented as mean ± standard deviation (SD) for normal distribution variables or as median and interquartile range (IQR) for non-normal distribution variables. Qualitative variables were reported as absolute numbers and percentages (%). The nonparametric Mann–Whitney U test was used for quantitative variables with non-normal distribution. Pearson’s correlation coefficient was calculated to evaluate variations in annual incidence and age at diagnosis over time. Confidence intervals for incidence rates were estimated assuming the Poisson distribution. All statistical analyses were per-formed with IBM SPSS Statistics for Windows, version 20.0 (IBM Corp, Armonk, NY, USA) and Stata 16/SE (Stata Corp, College Station, TX, USA).

This study was conducted according to the guidelines of the Declaration of Helsinki and approved by the Clinical Research Ethics Committee (ethical approval code 2020.083 and March 2020).

## 3. Results

### 3.1. Demographics and Clinical Features at Baseline

NBD was diagnosed in 23 of 96 (24%) cases. Fifteen women and eight men with a mean age of 44 ± 14 years were included in this study. HLA-B51 was positive in 5 of 13 (38.4%) patients tested. 

Parenchymal neurological involvement was observed in 10 (43.5%) patients, non-parenchymal damage in 10 (43.5%) patients, and 3 (13%) patients had mixed NBD. Parenchymal involvement was divided into multifocal/diffuse (*n* = 4, 40%), optic neuropathy (*n* = 3, 30%), cerebral (*n* = 2, 20%), and cerebellar involvement (*n* = 1, 10%) categories. All cases of non-parenchymal damage were acute meningeal syndrome (*n* = 10, 100%). Mixed phenotype neurological involvement included cerebellar injury and acute meningeal syndrome (*n* = 1), optic neuropathy and acute meningeal syndrome (*n* = 1), and cerebral and intracranial hypertension (*n* = 1). All demographic and clinical features are summarized in Table 1.

### 3.2. Organ Involvement

Other clinical domains of BD were present in all patients and were divided into mucocutaneous (*n* = 20, 87.0%), articular (*n* = 15, 65.2%), ocular (*n* = 14, 60.9%), cutaneous (*n* = 10, 43.5%), and vascular involvement (*n* = 4, 17.4%) categories. The frequency of other clinical domains was not significantly different between males and females, except mucocutaneous involvement (100% in females and 62.5% in males, *p* = 0.011) (Table 1).

### 3.3. Treatment Administered

A total of 16 (69.6%) patients received oral corticosteroids (a mean maximum dose of 42 ± 12.5 mg/day), 13 (56.5%) received conventional immunosuppressants, and 7 (30.4%) received biological therapy (BT). BT was used in patients who were refractory to conventional immunosuppressive agents. Anti-TNFα monoclonal antibodies were used as the first option in all patients who received BT. However, in three of seven (42.7%) patients, the BT had to be switched due to inefficacy. Table 2 shows the main NBD clinical subtypes and the therapeutic schemes for these patients.

### 3.4. Incidence by Age, Gender, and Year

**Figure 1:**
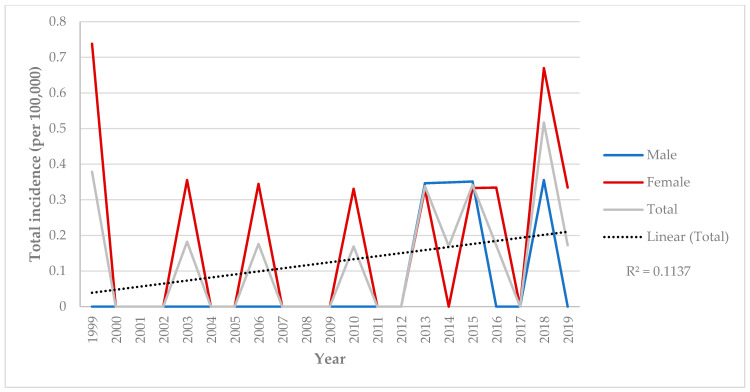
Incidence of Neuro-Behçet’s disease in residents of Santander Healthcare Area, Spain, in 1999–2019 according to gender.

The total annual incidence of NBD in Cantabria from 1999 to 2019 was 0.13 per 100,000 people (95% CI: 0.11–0.26). Specifically, the incidence was 0.14 per 100,000 men (95% CI: 0.04–0.23) and 0.24 per 100,000 women (95% CI: 0.12–0.37). Annual incidence rates varied, ranging from a minimum of 0.17 per 100,000 in 2010 to a maximum of 0.51 per 100,000 in 2018 (Figure 1).

**Figure 2:**
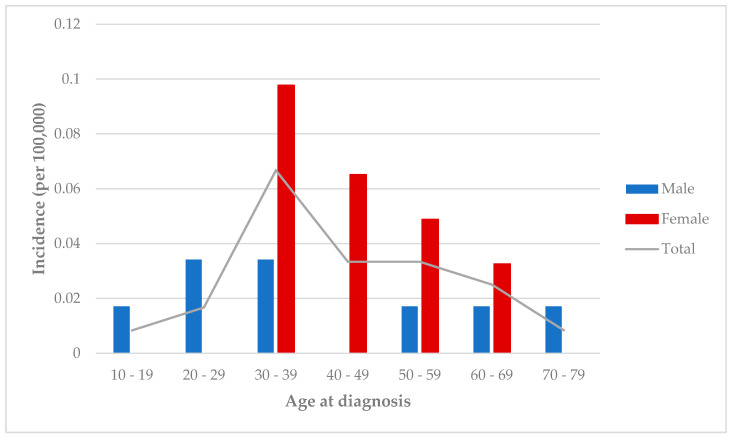
Incidence of Neuro-Behçet’s disease in Cantabria, Spain, from 1999 to 2019 stratified by age and gender.

The highest incidence rate was recorded in women aged 30–39 years (0.07 per 100,000 people) and men aged 20–39 years (0.03 per 100,000 people). Conversely, the lowest incidence rates were observed in the 10–19 years and 80–89 years age groups, with both showing a rate of 0.018 per 100,000 people (see Figure 2). When analyzing the incidence rates by age, no significant differences between genders were found (*p* > 0.05).

**Figure 3:**
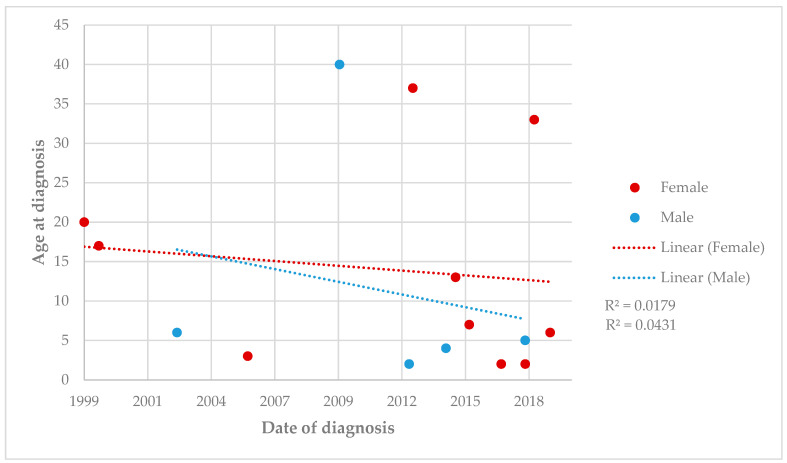
Trends in age at Neuro-Behçet’s disease diagnosis in Cantabria, Spain, in 1999–2019 by gender.

There was a linear relationship with a mild decrease in age at diagnosis over time. However, no significant correlation was found for either men (r = 0.04) or women (r = 0.02) (Figure 3).

## 4. Discussion

To the best of our knowledge, we present the most extensive Spanish series of patients with NBD. In this study, NBD was diagnosed in nearly a quarter of BD cases, with predominance in women, and above one-third of those tested were HLA-B51 positive. In addition, the annual incidence of NBD in our region during the study period (1999–2019) was 0.13 per 100,000 people/year.

The main primary studies that have analyzed NBD’s epidemiological aspects are summarized in Table 3. Furthermore, our findings are similar to those of other Spanish articles [2,11]. The only exception is the 2010 Spanish 20-year retrospective study in which 20 patients were diagnosed with NBD out of 360 patients with BD [12], a relatively low prevalence compared to other similar studies. That study included patients from more than 40 years ago. An increase in the detection rates of NBD due to improvements in diagnostic techniques may explain the higher frequency of NBD.

Interestingly, western Mediterranean and Middle Eastern countries seem to have a higher number of NBD diagnoses. For example, a 2013 Tunisian retrospective study, in which 121 patients were diagnosed with NBD out of 430 patients with BD, showed the highest percentages of frequency of NBD diagnosis [13]. On the other hand, the previously mentioned 2010 Spanish retrospective study (20 NDB/360 BD) and the 2020 Turkish retrospective article (26 NBD/419 BD) had the lowest percentages of frequency [14]. These findings may be due to geographic differences and the heterogeneity of clinical features of the disease, and may suggest a shift away from the historical perception of higher prevalence along the Silk Road [15,16]. In addition, high frequencies of Northern European alleles are observed in Cantabrians due to a unique genetic pool within the Iberian Peninsula [17]. This may explain NBD frequency in Cantabria in comparison to the rest of Spain [3,11,12]. 

In terms of gender and age variations, in contrast to our study, there seems to be a male predominance. Turkish (77.5%), Japanese (75%), and Iraqi (70%) retrospective studies showed the highest rates [8,18,19]. Gender predilection for BD has been controversial [20]. However, the male gender is a known risk factor for a more severe disease course in BD, such as NBD [20]. Our study shows similar results to other reports regarding age at the time of diagnosis: onset before the age of 15 years and diagnosis after the age of 50–55 years seems infrequent [20]. A 2015 Portuguese study (37.5 ± 9.4 years) and our study (44 ± 13.9 years) had the highest ages at diagnosis [21].

The presence of HLA-B51 was similar for BD and NBD in most revised studies. The only exception is a 2013 Tunisian retrospective study with a higher HLA-B51 positivity in NBD than in BD [13] (Table 3). HLA has been shown to influence the innate immune response and dysbiosis, both of which may play a crucial role in the development of BD [22,23]. It has been established that HLA-B51 has a modest effect on disease phenotype, which may explain similarities found in rates in different studies [20]. 

There seem to be similarities in epidemiological trends during the past two decades in the studies reviewed. However, there has been a tendency of lower frequencies in detection rates of NBD the older a study is. The 2003 Iraqi study (14.3%), the 2010 Japanese retrospective study (13%), and the 2010 Spanish retrospective study (5.6%) showed the lowest frequencies [12,19,24]. In contrast, the 2018 Tunisian retrospective study (28.1%) and the 2019 Spanish retrospective study (25.6%) had some of the highest frequencies [11,13]. These trends may be due to an increase in the detection rates of NBD due to improvements in diagnostic techniques. Nevertheless, no differences have been observed in age at diagnosis, for which most detection rates are in the third and fourth decade of life. For example, in the 2003 Iraqi retrospective study, patients were diagnosed with NBD at a mean age of 34.1, while in the 2019 Spanish study they were diagnosed at 31.8 years [8,11].

The present study has several limitations. Firstly, patients were mainly identified using diagnostic codes from the three hospitals’ databases. This may have introduced some level of misclassification or omission, potentially leading to the under-detection of certain cases, which may have resulted in an underestimation of true incidence rates. Secondly, the retrospective nature of this study may also underestimate the true incidence of NBD. Retrospective studies often depend on existing records, which may be incomplete or inconsistent, leading to potential biases in data. Moreover, reliance on historical records means that any changes in diagnostic practices, record-keeping, or patient management over time may influence findings. As a result, incidence rates observed in this study might not accurately reflect the actual occurrence of NBD in the broader patient population.

**Table 3:** Main clinical features and treatment of 23 patients with Neuro-Behçet’s disease.

Author, Year	Country	*n* Cases			Male, *n* (%)		Age at Onset, Years Mean ± SD		HLA-B51+, *n* (%)	
		BD	NBD	%	BD	NBD	BD	NBD	BD	NBD
E. Bolek et al., 2020 [15]	Turkey	419	26	6.2	225 (53.7)	39 (56.5)	29.2 ± 9.0	27.4 ± 9.2	104 (69.3)	13 (65)
Charca-Benavente et al., 2019 [11]	Spain	39	10	25.6	20 (51.3)	6 (60)	31.8 ± 13.9	29.7 ± 12.1	ND	ND
Riera-Maestra et. al., 2010 [12]	Spain	360	20	5.6	ND	13	34	36.3	ND	ND
Peño et al., 2012 [2]	Spain	25	7	28.0	11 (43)	4 (56)	32	29	ND	ND
Akman-Demir et al., 1999 [18]	Turkey	ND	200	ND	ND	155 (77.5)	ND	31.5	ND	ND
Houman et al., 2013 [12]	Tunis	430	121	28.1	295 (68.6)	78 (64.5)	29.17	29.02	84 (19.5)	14 (33.3)
Ideguchi et al., 2010 [19]	Japan	412	54	13	33 (61)	33 (75)	36.9 ± 11.9	35.8 ± 10.3	123 (50)	16 (55)
Talarico et al., 2012 [1]	Italy	117	13	38	72 (61.5)	36 (50)	25 ± 4	25 ± 4	77 (66)	ND
Al-Araji et al., 2003 [8]	Iraq	140	20	14.3	105 (75)	14 (70)	34.2	34.1	ND	ND
Domingos et al., 2015 [21]	Portugal	138	25	18.1	45 (32.6)	10 (40)	35.8 ± 9.2	37.5 ± 9.4	59 (42.9)	10 (41.1)
Present study, 2022	Spain	96	23	24	58 (60.4)	8 (34.8)	38 ± 13.9	44 ± 13.9	43 (48.3)	5 (38.4)

## 5. Conclusions

In summary, our results indicate that the epidemiological characteristics of NBD in Northern Spain are similar to those of other neighboring countries, except female gender predominance.

## Figures and Tables

**Table 1:** Demographic and clinical features of 23 patients with Neuro-Behçet’s disease in the 1999–2019 period in Santander, Spain, and differences according to gender.

Clinical Characteristics	Female (n = 15)	Male (n = 8)	Total (n = 23)	*p* Value
**Demographic parameters**				
- Age, years (**mean** ± SD)	46 ± 10	41 ± 18.5	44 ± 13.9	0.325
- HLA-B51 positive, n (%)	2/9 (22.2)	3/4 (75)	5/13 (38.4)	0.071
- Follow-up time (yrs), **median** [IQR]	17 [6.5–24.5]	6 [3.5–28.5]	13 [4.5–26.5]	0.506
**Non-neurological involvement, n (%)**				
- Mucocutaneous involvement	15 (100)	5 (62.5)	20 (83.3)	0.011
- Articular involvement	11 (73.3)	4 (50)	15 (65.2)	0.263
- Ocular involvement	9 (60)	5 (62.5)	14 (60.9)	0.907
- Skin lesions	6 (40)	4 (50)	10 (43.5)	0.645
- Vascular involvement	2 (13.3)	4 (50)	4 (17.4)	0.263
**Neurological involvement**				
**Parenchymal phenotype, n (%)**	8 (53.3)	2 (25)	10 (43.5)	0.192
- Multifocal/diffuse	3 (37.5)	1 (50)	4 (40)	0.651
- Optic neuropathy	3 (37.5)	0	3 (30)	0.175
- Cerebral involvement	1 (12.5)	1 (50)	2 (20)	0.455
- Cerebellar involvement	1 (12.5)	0	1 (10)	0.455
**Non-parenchymal phenotype, n (%)**	4 (26.7)	6 (75)	10 (43.5)	0.026
- Acute meningeal syndrome	4 (100)	6 (100)	10 (100)	0.026
**Mixed phenotype, n (%)**	3 (20)	0	3 (13)	0.175
- Cerebellar involvement and acute meningeal syndrome	1 (33.3)	0	1 (33.3)	0.455
- Optic neuropathy and acute meningeal syndrome	1 (33.3)	0	1 (33.3)	0.455
- Multifocal/diffuse and intracranial hypertension	1 (33.3)	0	1 (33.3)	0.455

**Table 2:** Treatment of 23 patients with Neuro-Behçet’s disease.

	*n* (%)	Mean Maximum Oral Prednisone Dose, (SD) mg/day	Conventional Immunosuppressants, *n* (%)	Monoclonal Anti-TNFα, *n* (%)	Tocilizumab, *n* (%)	Anakinra, *n* (%)
**Parenchymal phenotype**	10 (43.5)	51.7 ± 19.3	6 (46.2)	4 (54.1)	0	0
- Multifocal/diffuse	5 (50)	52.5 ± 7.5	2 (50)	3 (75)		
- Optic neuropathy	3 (30)	52.3 ± 26.3	2 (66.7)	0		
- Cerebral	1 (10)	45	1 (16.7)	0		
- Cerebellar	1 (10)	0	1 (16.7)	1 (25)		
**Non-parenchymal phenotype**	10 (43.5)	42 ± 12.5	5 (38.5)	2 (28.6)	0	0
- Acute meningeal syndrome	10 (43.5)	42 ± 12.5	5 (50)	2 (28.6)		
**Mixed**	3 (13)	45 ± 15	2 (15.4)	1 (100)	1 (100)	1 (100)
- Cerebellar involvement and acute meningeal syndrome	1 (33.4)	60	0	0	0	0
- Optic neuropathy and acute meningeal syndrome	1 (33.4)	0	1 (50)	0	0	0
- Cerebral and intracranial hypertension	1 (33.4)	30	1 (50)	1 (100)	1 (100)	1 (100)

## Data Availability

The original contributions presented in the study are included in the article, further inquiries can be directed to the corresponding author.

## Supplementary Materials

The following supporting information can be downloaded at: https://www.mdpi.com/article/10.3390/jcm13175270/s1, Figure S1. Location map of Cantabria region (red) in Spain.
