# Supplementary material for: Epidemiology of Neuro-Behçet’s Disease in Northern Spain 1999–2019: A Population-Based Study
Source: J Clin Med. 2024 Sep 5;13(17):5270. doi: 10.3390/jcm13175270 (PMC11395878; doi:10.3390/jcm13175270)
Supplement: Supplementary file 1 [file jcm-13-05270-s001.zip › jcm-3184606-supplementary.pdf]

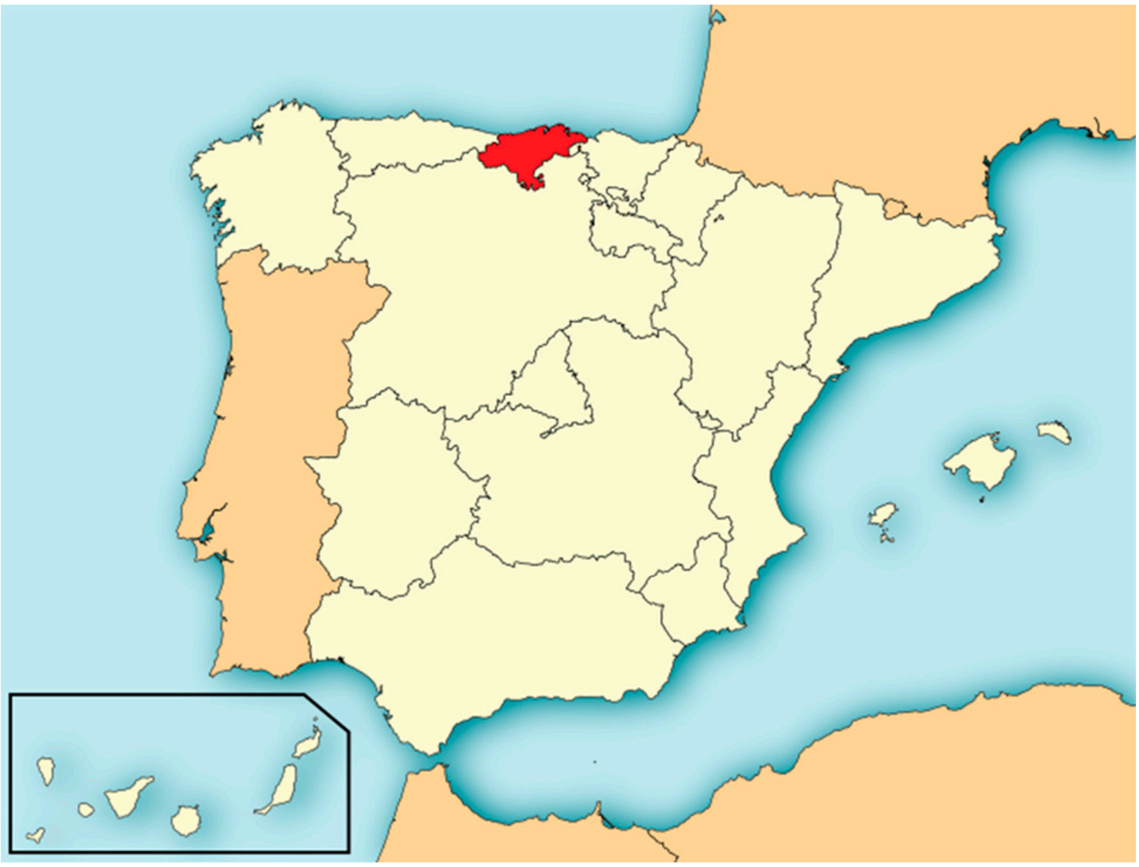

From Wikimedia Commons, the free media repository

Figure S1. Location map of Cantabria region (red) in Spain.
